# Multimodal Retinal Imaging for Detection of Ischemic Stroke

**DOI:** 10.3389/fnagi.2021.615813

**Published:** 2021-02-02

**Authors:** Lu Zhao, Hui Wang, Xiufen Yang, Bin Jiang, Hongyang Li, Yanling Wang

**Affiliations:** ^1^Department of Ophthalmology, Beijing Friendship Hospital, Capital Medical University, Beijing, China; ^2^Department of Ophthalmology, Beijing Chao-Yang Hospital, Capital Medical University, Beijing, China; ^3^Department of Neurology, Beijing Friendship Hospital, Capital Medical University, Beijing, China; ^4^Multidisciplinary Team Center for Ocular Vascular Diseases, College of Ophthalmology, Capital Medical University, Beijing, China

**Keywords:** ischemic stroke, infraction, prediction, retinal, subfoveal choroidal thickness, ocular blood flow

## Abstract

**Background**: This study aims to evaluate ocular changes in patients with ischemic stroke using multimodal imaging and explore the predictive value of ocular abnormalities for ischemic stroke.

**Methods**: A total of 203 patients (ischemic stroke group, 62; control group, 141) were enrolled in this study. Basic data from patients, including age; gender; height; weight; history of hypertension, hyperlipidemia, diabetes, alcohol use, and coronary heart disease; and smoking status, were collected. Consequently, Doppler color ultrasound, color fundus photography, and optical coherence tomography (OCT) examinations were conducted. Differences in traditional risk factors and ocular parameters between the two groups were compared, and binary logistic regression was used for multivariate analysis.

**Results**: The central retinal artery equivalent (CRAE) in the ischemic stroke group was 150.72 ± 20.15 μm and that in the control group was 159.68 ± 20.05 μm. The difference was statistically significant (*P* = 0.004). Moreover, the subfoveal choroidal thickness (SFChT) in the ischemic stroke group was 199.90 ± 69.27 μm and that in the control group was 227.40 ± 62.20 μm. The difference was statistically significant (*P* = 0.006). Logistic regression results showed that smoking [odds ratio (OR) = 2.823; 95% confidence interval (95% CI) = 1.477–5.395], CRAE (OR = 0.980; 95% CI = 0.965–0.996), and SFChT (OR = 0.994; 95% CI = 0.989–0.999) are associated with increased risk of ischemic stroke when ocular parameters were combined with traditional risk factors. The area under the receiver operating characteristic (ROC) curve was 0.726, which shows good diagnostic accuracy.

**Conclusion**: SFChT may be a diagnostic marker for early detection and monitoring of ischemic stroke. Combined with traditional risks, retinal artery diameter, and choroidal thickness, the prediction model can improve ischemic stroke prediction.

## Introduction

Around 25.7 million survivors of stroke exist worldwide. Most strokes (71%) are ischemic, which is the most common type of stroke (Meschia and Brott, 2018). Moreover, retinal blood vessels are the only blood vessels that can be directly observed in living subjects. They share the same origin as cerebral blood vessels and are thus similar in anatomical structure, physiological metabolism, and pathological changes. Previous studies have shown that abnormalities in retinal blood vessels can be a *window* for effective evaluation of cerebral vasculopathy (London et al., 2013; Zafar et al., 2019). Furthermore, color fundus photographs were produced in the 1950s and have become one of the main examination methods for retinopathy screening because it is simple, fast, economical, and noninvasive. Abnormalities in retinal blood vessels can be directly observed in color fundus photographs, including arteriolar narrowing (focal or generalized), arteriovenous nicking, microaneurysms, hemorrhages, exudates, cotton–wool spots, and edema. With the development of digital image processing, image analysis software can be used to quantitatively measure retinal blood vessel parameters such as central retinal artery equivalent (CRAE), central retinal vein equivalent (CRVE), and arteriovenous ratio (AVR). The results of population-based prospective or cross-sectional studies (e.g., the Atherosclerosis Risk in Communities Study, Beaver Dam Eye Study, Blue Mountains Eye Study, and Singapore Malay Eye Study) showed that retinal microvascular abnormalities found in fundus photographs are associated with stroke (Klein et al., 1999; Wong et al., 2001; Mitchell et al., 2005; Cheung et al., 2013). In recent years, the retinal vascular caliber has been independently correlated with stroke (Hughes et al., 2016; Seidelmann et al., 2016; McGrory et al., 2019; Rim et al., 2020). Thus, retinal vascular caliber measurements are one of the important markers for stroke (Kipli et al., 2018).

Color Doppler flow imaging (CDFI) is a quantitative, direct, noninvasive, and reproducible examination method for retrobulbar blood flow. Moreover, CDFI is widely used in many ophthalmological diseases, particularly ischemic eye or ocular diseases (Dimitrova and Kato, 2010; Pauk-Domańska and Walasik-Szemplińska, 2014; Abegão Pinto et al., 2016; Jianu et al., 2016; Bienert et al., 2018; Kuerten et al., 2019; Kilic et al., 2020; e.g., diabetic retinopathy, anterior ischemic optic neuropathy, retinal artery occlusion, retinal vein occlusion, ocular ischemic syndrome, glaucoma, and central serous chorioretinopathy). Similarly, this test method can be used to evaluate the retrobulbar blood flow in patients with ischemic stroke.

Spectral-domain optical coherence tomography (OCT) and enhanced-depth imaging are undergoing continuous development. Both can be used for scanning choroid cross-sections and quantitatively measuring choroid thickness. The examination process is non-invasive, rapid, and highly reproducible, with promising clinical application. Choroid thickness has proven to be important in the diagnosis of various ophthalmologic and systemic diseases (Tan et al., 2016; Obis et al., 2018; Steiner et al., 2019; Torabi et al., 2019). Currently, the relationship between choroid thickness and disease is a research hotspot.

Optic fundus abnormalities have opened a window for studying stroke. Previous studies found that retinal vascular caliber was associated with ischemic stroke. However, the retinal imaging software is not of routine use due to the limitations of the methods in predicting stroke. The current study employed ophthalmologic multimodal imaging for the integrated assessment of various ocular examination parameters to discover a method for efficient and sensitive detection of ischemic stroke, thereby improving stroke risk prediction.

## Materials and Methods

Data supporting the findings of this study are available from the first author on reasonable request.

### Study Design and Participants

This study is an observational cross-sectional study. Between January 2016 and January 2018, 62 patients with confirmed ischemic stroke and 141 comparison patients with no stroke were recruited from Beijing Friendship Hospital, Capital Medical University, Beijing, China. This study was approved by the ethics committee of Beijing Friendship Hospital, Capital Medical University (approval code: 2018-P2-209-02), and conformed to the *Declaration of Helsinki (2013 revised edition)*. All patients voluntarily participated in this study and signed the informed consent form.

### Inclusion Criteria

Patients aged ≥50 years were diagnosed with ischemic stroke at the research hospital and received inpatient care. Diagnoses were based on the 2014 Chinese Guidelines for the Diagnosis and Treatment of Acute Ischemic Stroke (Chinese Society of Neurology and Chinese Stroke Society, 2015). Of the five subtypes of acute ischemic stroke by the Trial of ORG 10172 in Acute Stroke Treatment (TOAST) classification (Adams et al., 1993), large-artery atherosclerosis and small-vessel occlusion subtypes were included. However, cardioembolism, a stroke of other determined etiology, and a stroke of undetermined etiology subtypes were excluded. All enrolled patients had a stroke that occurred 6–12 months before enrollment.

The control group consisted of patients with no stroke aged ≥50 years during the same period in whom cerebral magnetic resonance imaging (MRI) results showed no old or new cerebral infarction or no obvious abnormality in computed tomography (CT). All patients with no stroke denied the history and symptoms of a stroke. Patients with cerebral CT/MRI intracerebral hemorrhage and intracranial space-occupying lesions were excluded.

### Exclusion Criteria

The exclusion criteria were patients in whom clear optic fundus images could not be obtained; patients who did not cooperate with various ophthalmological examinations; and patients with ocular or systemic diseases (e.g., anterior segment lesions or refractive media turbidity that affects fundoscopy such as severe cataracts and corneal lesions), active ocular inflammation or infection (e.g., uveitis), glaucoma, pathological myopia, comorbid retinopathy (e.g., diabetic retinopathy, senile macular degeneration, polypoidal choroidal vasculopathy, retinal artery occlusion, retinal vein occlusion, and retinal pigmentary degeneration), optic neuropathy (e.g., optic neuritis) and anterior ischemic optic neuropathy, any history of retinal surgery including laser therapy, and systemic infection or tumors.

### Examinations

Basic data from patients, which include age; gender; height; weight, history of hypertension, hyperlipidemia, diabetes, coronary heart disease, alcohol use (average, >50 g each day; duration, at least 1 year); and current or previous smoking status (average, ≥1 cigarette/day; duration, at least 1 year), were collected based on risk factors for stroke that were reported in the literature. Hypertension was defined as systolic blood pressure of ≥140 mmHg (1 mmHg = 0.133 kPa), diastolic blood pressure of ≥90 mmHg, or past definitive diagnosis of hypertension and currently on antihypertensive medicine. Moreover, a history of hyperlipidemia and diabetes was defined as a medical history record of treatment or a definitive diagnosis of hyperlipidemia and diabetes after inpatient treatment for stroke. A history of coronary heart disease was defined as a history of angina pectoris or myocardial infarction. Furthermore, all patients underwent routine ophthalmologic slit-lamp examination, color Doppler ultrasound examination of the eye, color fundus photography after mydriasis, and OCT examination. Ipsilateral eyeball measurement parameters were selected from patients with ischemic stroke. In the healthy control group, right eye measurement parameters were included for statistical analysis.

### Color Doppler Ultrasound of Retrobulbar Blood Flow

**Figure 1:**
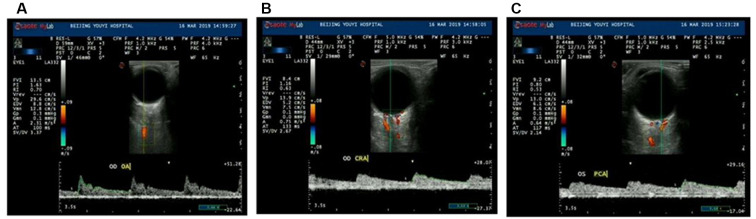
Ultrasound Doppler examinations of ocular vessels and the measurement process of major vascular parameters. The parameters are as follows: peak systolic velocity (PSV is shown as Vp), end-diastolic velocity (EDV), resistance indices (RI), and pulsatility index (PI). The images demonstrate measurements of the ophthalmic artery (OA; **A**), central retinal artery (CRA; **B**), and posterior ciliary artery (PCA; **C**).

The retrobulbar blood flow was measured using an ultrasound system (MyLab Class C, Genoa, Italy) with a probe frequency of 4–12 MHz, a sampling volume of 1 mm, and the angle between the sonic beam and blood vessel adjusted to <20°. The subject adopted a supine position with eyes closed. The probe was used to gently contact the upper eyelid of the subject (without exerting pressure on the eyeball) for scanning. Consequently, CDFI was performed for the peak systolic velocity (PSV), end-diastolic velocity (EDV), pulsatility index (PI), and resistance index (RI), which were measured for retrobulbar vessels, including the ophthalmic artery (OA), central retinal artery, and posterior ciliary artery (PCA; Figure 1). The best images in 3 to 5 adjacent cardiac cycles were selected. All measurements were taken by the same experienced technician.

### Color Fundus Photography and Retinal Vascular Caliber Measurement

A fundus camera (Kowa Company, Ltd., Osaka, Japan) was used for color fundus photography after bilateral mydriasis. Photographs were taken with the midpoint of the macula as the center and 45° around the center. Color fundus photographs included the optic disc, macula, vascular arches at the temporal macula, and nasal regions at two optic disc diameters from the edge of the optic disc. All photograph acquisition was completed by the same qualified technician.

**Figure 2:**
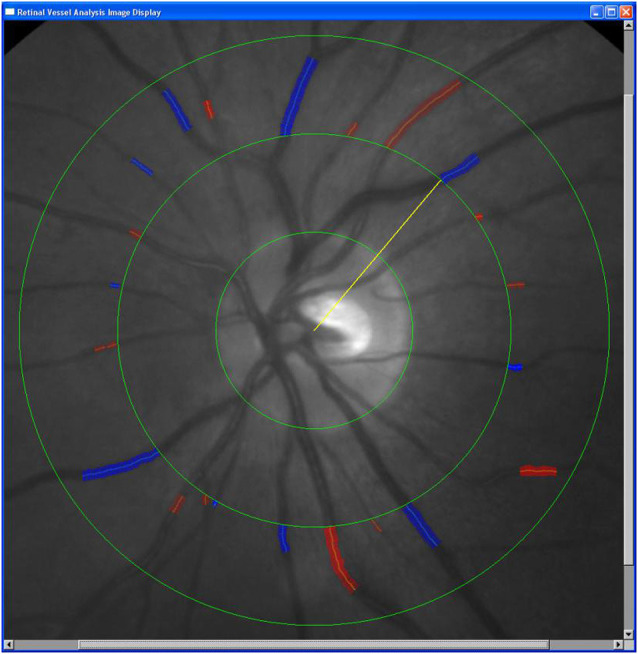
Retinal arteriolar and venular diameters. Semiautomated software (IVAN software, Sydney, NSW, Australia) was used for quantitative analysis. The software employs automatic optic disc detection and measures the caliber of six peripheral retinal arteriolar and venular vessel diameters up to 1 disc diameter (DD) from the optic disc margin. The three rings from the inside to the outside are the Disc diameter ring, 1/ 2DD ring from the optic disc margin, and the 1DD ring from the optic disc margin. The red and blue mark delicate retinal artery and retinal vein, respectively, and the yellow line is the marking line used to measure the vessel diameter.

Retinal vascular caliber was measured by a semiautomated software (IVAN software, Sydney, NSW, Australia). The standardized measurement protocols have been previously described (Hubbard et al., 1999; Sherry et al., 2002;). The computer-based software automatically measures blood vessel diameters of retinal arteries and veins at 1/2 − 1 disc diameter (DD) from the optic disc margin (Figure 2). The formula of Knudtson et al. (2003) for calculating the relationship of retinal blood vessel branches and trunk diameter was used to obtain the primary parameters CRAE, CRVE, and AVR.

### Optical Coherence Tomography (OCT) and Choroid Thickness Measurement

**Figure 3:**
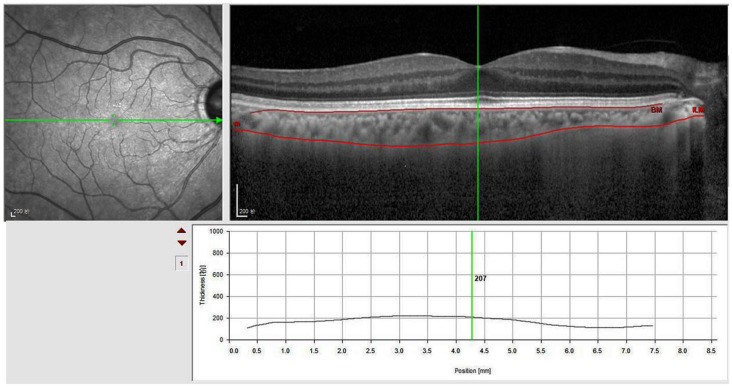
Subfoveal choroidal thickness (SFChT) measurements. Using the analysis software of OCT, manually segmented internal limiting membrane line to the choroidoscleral junction. When we adjusted the vertical line perpendicular to the center of the fovea, SFChT was calculated automatically.

The choroid was scanned using enhanced-depth imaging spectral-domain OCT (Spectralis; Heidelberg Engineering, Heidelberg, Germany). The default measurement tool of the OCT machine was used to manually measure the subfoveal choroidal thickness (SFChT). SFChT is defined as the vertical distance from the hyperreflective line of the Bruch membrane to the hyperreflective line of the inner surface of the sclera (Figure 3). Consequently, OCT examinations were conducted by the same experienced technician. Choroid measurements were blindly conducted by two retinopathy experts.

### Statistical Analysis

The Statistical Package for the Social Sciences software, version 26.0, was used for the statistical analysis of the data. Quantitative data were expressed as mean (± standard deviation). The independent sample *t*-test and rank-sum test were used if data were normally and not normally distributed, respectively. Consequently, Fisher’s exact and chi-square tests were used for qualitative data. Bivariate logistic regression was used to analyze the relationship between traditional risk factors and ischemic stroke (model 1), the relationship between ocular parameters and ischemic stroke (model 2), and parameters with statistically significant differences in models 1 and 2 that were included in logistic regression (model 3). A difference of *P* < 0.05 was considered statistically significant. The area under the receiver operating characteristic (ROC) curve was used to evaluate the accuracy of the regression model diagnosis.

## Results

### Baseline Information of Patients

Overall, 203 patients (ischemic stroke group, 62; control group, 141) were enrolled in this study. Table 1 shows the baseline information of patients. No statistically significant differences were noted in age; gender; BMI; and history of diabetes, hypertension, hyperlipidemia, and coronary heart disease between the two groups. However, statistically, significant differences were observed in smoking status and alcohol use history (Table 1). Consequently, the 203 patients were divided into two groups: smokers and nonsmokers. Moreover, no significant difference was observed in SFChT between the two groups (*P* = 0.910).

**Table 1:** Baseline characteristics of patients.

	Control	Ischemic stroke	*P*-value
Age (years)	62.69 ± 7.47	64.76 ± 7.36	0.069
BMI (kg/m^2^)	25.47 ± 3.56	25.28 ± 3.51	0.720
Sex			0.190
Male (%)	52 (36.9%)	17 (27.4%)
Female (%)	89 (63.1%)	45 (72.5%)
Hypertension			0.247
Absent	38 (26.9%)	12 (19.4%)
Present	103 (73.1%)	50 (80.6%)
Hyperlipidemia			0.408
Absent	68 (48.2%)	26 (41.9%)
Present	73 (51.8%)	36 (58.1%)
Diabetes			0.105
Absent	92 (65.2%)	33 (53.2%)
Present	49 (34.8%)	29 (46.8%)
Coronary heart disease			0.350
Absent	117 (82.9%)	48 (77.4%)
Present	24 (17.1%)	14 (22.6%)
Smoking			0.001
Absent	91 (64.5%)	25 (40.3%)
Present	50 (35.4%)	37 (59.7%)
Alcohol drinking			0.024
Absent	92 (65.2%)	30 (48.4%)
Present	49 (34.8%)	32 (51.6%)

### Ocular Multimodal Imaging Parameters

In CRAE, SFChT decreased in the ischemic stroke group compared with the control group, and these differences were statistically significant. Other parameters did not show statistically significant differences (Table 2).

**Table 2:** Comparison of ocular parameters between the two groups.

	Control	Ischemic stroke	*P*-value
CRAE (μm)	159.68 ± 20.05	150.72 ± 20.15	0.004
CRVE (μm)	242.92 ± 26.45	242.81 ± 23.88	0.977
AVR	0.66 ± 0.08	0.64 ± 0.08	0.050
SFChT (μm)	227.40 ± 62.20	199.90 ± 69.27	0.006
OA			
PSV (cm/s)	35.06 ± 15.75	33.86 ± 10.45	0.521
EDV (cm/s)	8.70 ± 3.91	7.81 ± 3.99	0.142
RI	0.74 ± 0.10	0.77 ± 0.08	0.082
PI	1.19 ± 0.24	1.26 ± 0.20	0.050
CRA			
PSV (cm/s)	11.53 ± 3.97	11.09 ± 3.92	0.473
EDV (cm/s)	3.68 ± 1.44	3.63 ± 1.56	0.822
RI	0.67 ± 0.09	0.67 ± 0.08	0.904
PI	1.03 ± 0.20	1.02 ± 0.18	0.790
PCA			
PSV (cm/s)	14.36 ± 4.45	14.67 ± 4.79	0.279
EDV (cm/s)	4.92 ± 2.41	4.66 ± 1.96	0.860
RI	0.66 ± 0.08	0.68 ± 0.07	0.083
PI	1.00 ± 0.19	1.05 ± 0.15	0.113

*CRAE, central retinal artery equivalent; CRVE, central retinal vein equivalent; AVR, arterio-venous ratio; SFChT, subfoveal choroidal thickness; OA, ophthalmic artery; CRA, central retinal artery; PCA, posterior ciliary artery; PSV, peak systolic velocity; EDV, end diastolic velocity; RI, resistance index; PI, pulsatility index*.

### Logistic Regression Model and Receiver Operating Characteristic Curve Analysis

**Figure 4:**
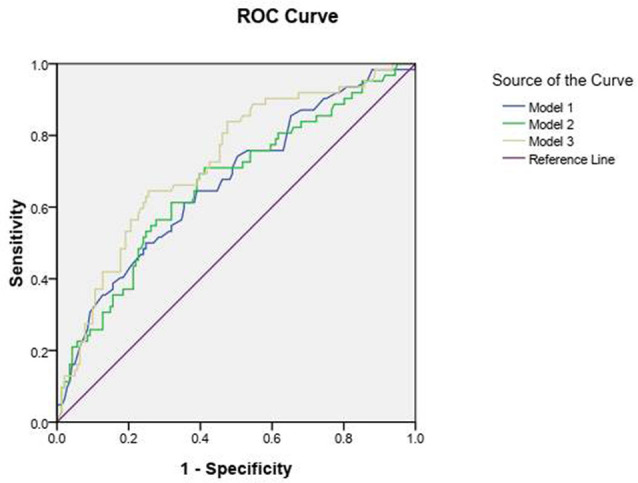
Receiver Operating Characteristic (ROC) curve analysis of Model 1, Model 2, and Model 3.

In model 1, common risk factors of ischemic stroke were used for logistic regression. The results showed that age (odds ratio [OR] = 1.046; 95% confidence interval [95% CI] = 1.000–1.092) and smoking (OR = 2.647; 95% CI = 1.096–6.390) were significantly associated with ischemic stroke (Table 3). Moreover, the area under the ROC curve was 0.668 (*P* = 0.000; 95% CI = 0.587–0.749; Figure 4).

**Table 3:** Logistic regression of ischemic stroke with risk factors or/and ocular parameters.

	B	S.E.	OR (95%CI.)	*P*-value
Model 1				
Age	0.045	0.022	1.046 (1.002, 1.092)	0.038
Smoking	0.973	0.450	2.647 (1.096, 6.390)	0.030
Constant	−4.220	1.448	0.015	0.004
Model 2				
CRAE	−0.021	0.008	0.979 (0.964, 0.995)	0.008
SFChT	−0.006	0.002	0.994 (0.989, 0.998)	0.011
Constant	3.775	1.323	43.604	0.004
Model 3				
CRAE	−0.020	0.008	0.980 (0.965, 0.996)	0.015
SFChT	−0.006	0.003	0.994 (0.989, 0.999)	0.031
Smoking	1.038	0.330	2.823 (1.477, 5.395)	0.002
Constant	1.218	2.139	3.380	0.569

*Model 1: logistic regression of ischemic stroke with traditional risk factors; Model 2: logistic regression of ischemic stroke with ocular parameters; Model 3: logistic regression of ischemic stroke with traditional risk factors and ocular parameters. CRAE, retinal artery equivalent; SFChT, subfoveal choroidal thickness*.

In model 2 on ocular parameters, the logistic regression model showed that CRAE (OR = 0.979; 95% CI = 0.964–0.995) and SFChT (OR = 0.994; 95% CI = 0.989–0.998) were associated with an increased risk of ischemic stroke. The area under the ROC curve was 0.664 (*P* *=* 0.000; 95% CI = 0.581–0.747; Figure 4).

In model 3, in which ocular parameters and traditional risk factors were combined, the logistic regression results showed that smoking (OR = 2.823; 95% CI = 1.477–5.395), CRAE (OR = 0.980; 95% CI = 0.965–0.996), and SFChT (OR = 0.994; 95% CI = 0.989–0.999) were associated with increased risk of ischemic stroke (Table 3). The area under the ROC curve was 0.726 (*P* *=* 0.000; 95% CI = 0.651–0.801; Figure 4).

## Discussion

This study examined known risk factors for ischemic stroke (Boehme et al., 2017), including unchangeable factors (age and gender) and factors amenable to intervention (BMI, hypertension, hyperlipidemia, diabetes, smoking, and alcohol use). The results showed that statistically significant differences exist in smoking status and alcohol use history. After adjusting for age, the logistic regression model showed that the risk of ischemic stroke was increased in smokers compared with nonsmokers (OR = 2.647; 95% CI = 1.096–6.390). This model showed some diagnostic accuracy because this was constructed based on traditional risk factors (area under the ROC curve = 0.668, *P* = 0.000; 95% CI = 0.587–0.749). Moreover, this study also examined ocular multimodal imaging parameters to further improve the accuracy of a stroke diagnosis by combining ocular parameters.

Since the 1990s, a large volume of population-based prospective or cross-sectional studies has proved that optic fundus changes are significantly associated with stroke (Klein et al., 1999; Wong et al., 2001; Mitchell et al., 2005). Subsequently, the correlation between retinal vascular caliber and stroke has been extensively studied with improvements in fundus imaging and evaluation technology. However, the measurement results are inconsistent. Prospective studies with a large sample size reported that retinal artery caliber is not associated with stroke, while increased retinal venular caliber is significantly associated with stroke (Ikram et al., 2006; Wong et al., 2006). Moreover, systematic reviews have also emphasized that the widening of retinal venular caliber rather than the narrowing of retinal artery caliber predicted stroke (McGeechan et al., 2009). Hughes et al. (2016) found that the narrowing of retinal artery caliber and lower AVR were associated with stroke. A study based on ARIC data showed that narrower retinal arterioles and wider retinal venules were associated with long-term risk of ischemic stroke (Seidelmann et al., 2016). Furthermore, Yatsuya et al. (2010) conducted a study based on ARIC data and found that narrower CRAE and wider CRVE were predictive of lacunar stroke. This study found a statistically significant difference in retinal artery caliber between the two groups, and retinal artery caliber showed a negative correlation with ischemic stroke. There are two possible reasons for these findings. First, the causes of stroke are complex and diverse, and many classification systems exist, while different subtypes of stroke are associated with different retinal abnormalities (Doubal et al., 2009; Dumitrascu et al., 2018). Large-artery atherosclerosis and small-vessel occlusion are the most common subtypes of TOAST classification. The two subtypes have many of the same risk factors (e.g., hypertension, diabetes, and smoking). The limitation of TOAST classification has been exposed to the development of imaging technology in recent years after these pathological subtypes have been diagnosed by high-resolution MR, digital subtraction angiography, and pathological examinations. For example, the diagnostic criteria of small-vessel occlusion are too loose to consider the different pathological changes of this subtype. Many cases that were previously considered as small-vessel occlusion subtypes should be identified as large-artery atherosclerosis subtypes (Gao et al., 2011; Chen et al., 2012; Zhang et al., 2019). Considering the aforementioned reasons, this study included these two subtypes. Second, small-artery sclerosis precedes aortic sclerosis. In this study, patients with ischemic stroke with aortic atherosclerosis and small-artery occlusion were enrolled. Therefore, it was deduced that the proportion of OA sclerosis is higher. The parameter of retinal artery caliber has more statistical significance because it can indirectly reflect vascular sclerosis.

The blood flow parameters for orbital blood vessels include PSV, EDV, RI, and PI. PSV reflects vascular congestion and blood flow supply, while EDV reflects blood perfusion in distal tissues. Thus, an increased PI suggests that blood flow is decreased during the diastolic phase and that vascular resistivity is increased. Multimodal eye examinations, including CDFI, were used to comprehensively evaluate the eye condition of patients with stroke. The results of CDFI showed that the difference between the two groups was not statistically significant, although the PSV and EDV of the OA in the stroke group were lower than those in the control group and the RI and PI increased.

Systemic diseases tend to affect choroid because it is rich in blood vessels. Therefore, choroid changes can be studied to understand systemic diseases. Moreover, studies have reported that choroid thickness has diagnostic and follow-up value for neurological diseases such as Alzheimer’s disease and migraine. Previous studies have reported choroidal thinning in ocular and systemic ischemic diseases (Altinkaynak et al., 2014; Kim et al., 2015; Zengin et al., 2015). A study on cerebral autosomal dominant arteriopathy with subcortical infarcts and leukoencephalopathy (CADASIL) reported that the mean SFChT in patients with CADASIL is significantly lower than that in the control group (Fang et al., 2017). In this study, SFChT was thinner compared with the control group, and the differences were statistically significant. The result may be explained with the possible mechanism. Retinal artery caliber became thinner and sclerotic in the ischemic stroke group. Consequently, choroid vessels could also be affected by atherosclerosis. Smoking was considered to be a possible factor that affects choroidal thickness. However, a meta-analysis showed that no significant effect of tobacco smoking on choroidal thickness change was detected (Yang et al., 2019). Furthermore, it was noted in this study that no significant difference exists in SFChT between smokers and nonsmokers. Logistic regression results showed that SFChT is associated with an increased risk of ischemic stroke. This suggests that SFChT may be an imaging biomarker to predict ischemic stroke.

This study combined traditional risk factors and ocular multimodal imaging parameters to construct a logistic regression model to predict ischemic stroke occurrence. This model has better diagnostic accuracy than a single risk factor or a single ocular parameter model as the area under the ROC curve is increased to 0.726.

However, this study has some limitations. First, this is a single-center cross-sectional study. Second, there may be a selection bias in enrolled patients. The patients with ischemic stroke are inpatients, and their condition may be relatively serious. Third, the sample size was relatively small and needs to be further enlarged.

## Conclusions

The decrease of CRAE and lower SFChT are associated with an increased risk of ischemic stroke. A prediction model based on the traditional risk factor of ischemic stroke with ocular changes can be used as a risk stratification tool to improve risk prediction.

## Data Availability Statement

The original contributions presented in the study are included in the article, further inquiries can be directed to the corresponding author/s.

## Ethics Statement

This study was approved by the ethics committee of Beijing Friendship Hospital, Capital Medical University (approval code: 2018-P2-209-02), and conformed to the Declaration of Helsinki (2013 revised edition). All patients voluntarily participated in this study and signed the informed consent form.

## Author Contributions

LZ and HL have conceived and designed the study. LZ contributed to the analysis, interpretation of data as well as drafting the manuscript and revising it critically. YW revised the manuscript and provided the final version to be published. HW, XY, and BJ were responsible for the acquisition of data. All the authors read and approved the final version of the manuscript.

## Conflict of Interest

The authors declare that the research was conducted in the absence of any commercial or financial relationships that could be construed as a potential conflict of interest.

## Abbreviations

AVR: arteriovenous ratio
CDFI: color Doppler flow imaging
CRAE: central retinal artery
CRAE: central retinal artery equivalent
CRVE: central retinal vein equivalent
CT: computed tomography
EDV: diastolic velocity
MRI: magnetic resonance imaging
OA: ophthalmic artery
OCT: optical coherence tomography
PCA: posterior ciliary artery
PI: pulsatility index
PSV: peak systolic velocity
RI: resistance index
ROC: receiver operating characteristic
SFChT: subfoveal choroidal thickness.
